# A Novel *Paludibacterium* Species Isolated from Human Blood

**DOI:** 10.3390/microorganisms14020280

**Published:** 2026-01-25

**Authors:** Akihiro Nakamura, Jun Murakami, Hitoshi Itohara, Tamaki Orita, Saori Ishimura, Misako Ohkusu, Kiyofumi Ohkusu, Masaru Komatsu

**Affiliations:** 1 Department of Clinical Laboratory Science, Faculty of Health Care, Tenri University, Tenri 632-0018, Nara, Japan; 2 Clinical Laboratory, Takarazuka City Hospital, Takarazuka 665-0827, Hyogo, Japan; 3 Department of Orthopaedic Surgery, Takarazuka City Hospital, Takarazuka 665-0827, Hyogo, Japan; 4 Medical Mycology Research Center, Chiba University, Chiba 263-8522, Japan; 5 Department of Microbiology, Tokyo Medical University, Tokyo 160-8402, Japan

**Keywords:** Neisseriales, Chromobacteriaceae, *Paludibacterium flexuosum* sp. nov., human blood, novel species

## Abstract

A novel facultatively anaerobic, Gram-negative, curved rod-shaped bacterium was isolated from blood cultures obtained from a patient with pyogenic spondylitis in Japan. The organism was additionally detected by 16S rRNA gene sequence analysis in a formalin-fixed, paraffin-embedded lumbar spine biopsy specimen from the same patient. The type strain, designated THUN1379ᵀ, is motile by means of a single polar flagellum and forms circular, white to translucent colonies on R2A agar and 5% sheep blood agar. The strain is oxidase-positive and catalase-negative and grows at temperatures ranging from 25 to 42 °C. Phylogenetic analysis based on the 16S rRNA gene sequence placed strain THUN1379ᵀ within the genus *Paludibacterium* (family Chromobacteriaceae), showing the highest sequence similarity to *Paludibacterium purpuratum* KJ031ᵀ (98.6%). Whole-genome sequencing revealed a genome size of approximately 3.6 Mb with a DNA G+C content of 61.3 mol%. Phylogenomic analysis based on whole-genome sequences supported the distinct taxonomic position of strain THUN1379ᵀ within the genus. The average nucleotide identity (ANI) and digital DNA-DNA hybridization (dDDH) values between strain THUN1379ᵀ and *Paludibacterium purpuratum* KJ031ᵀ were 79.7% and 23.1%, respectively, which are well below the accepted thresholds for species delineation. On the basis of phenotypic, chemotaxonomic, and genomic characteristics, strain THUN1379ᵀ represents a novel species of the genus *Paludibacterium*, for which the name *Paludibacterium flexuosum* sp. nov. is proposed. The type strain is THUN1379ᵀ (=JCM 36560ᵀ = KCTC 8465ᵀ).

## 1. Introduction

The genus *Paludibacterium*, belonging to the family Chromobacteriaceae within the order Neisseriales, was first proposed by Kwon et al. with the description of *Paludibacterium yongneupense*, isolated from a freshwater wetland in Korea [1]. At present, four species have been described within this genus: *P. yongneupense*, “*P. denitrificans*”, *P. paludis*, and *P. purpuratum* [1,2,3,4]. Members of the genus *Paludibacterium* are characterized as Gram-negative, facultatively anaerobic, non-spore-forming, curved rod-shaped bacteria that are motile by means of a single polar flagellum and are typically oxidase- and catalase-positive.

All previously described *Paludibacterium* species have been isolated exclusively from environmental sources, such as wetlands, marshes, and soil ecosystems, suggesting an ecological niche primarily associated with aquatic or terrestrial environments. To date, there have been no reports of *Paludibacterium* species isolated from human clinical specimens, and their potential clinical relevance remains unexplored.

During routine clinical microbiology laboratory testing, a previously uncharacterized *Paludibacterium*-like strain, designated THUN1379ᵀ, was isolated from blood cultures obtained from a patient diagnosed with pyogenic spondylitis; all four blood culture bottles were positive. Although the organism could not be isolated by culture from the lumbar spine biopsy specimen, its presence was subsequently confirmed by 16S rRNA gene sequence analysis performed on formalin-fixed, paraffin-embedded (FFPE) tissue from the same lesion.

In this study, we propose a novel species of the genus *Paludibacterium*, for which the name *Paludibacterium flexuosum* sp. nov. is proposed. To clarify its phylogenetic position and taxonomic distinctiveness, a polyphasic approach was employed, integrating phenotypic and biochemical characterization, MALDI-TOF mass spectrometry profiling, 16S rRNA gene sequence analysis, and whole-genome-based comparative analyses.

## 2. Materials and Methods

### 2.1. Isolation and Cultivation

Strain THUN1379^T^ was isolated in September 2020 from a blood culture of a 78-year-old male patient with pyogenic spondylitis at Takarazuka City Hospital, Japan. Ethical approval for the study was granted by the institutional review board (Approval number: 20210202; Approval date: 2 February 2021). In addition, the same strain was detected retrospectively from a formalin-fixed, paraffin-embedded lumbar spine biopsy specimen by 16S rRNA gene sequencing, although isolation from this specimen was unsuccessful due to prior antimicrobial therapy. The strain was cultured aerobically on tryptic soy agar (TSA) and 5% sheep blood agar (Becton Dickinson, Franklin Lakes, NJ, USA) at 35 °C under 5% CO_2_ for 24 h. Colony morphology was observed after 24 h of incubation.

### 2.2. Phenotypic Characterization

Bacterial morphology and Gram staining were examined using a standard Gram-staining kit (Muto Pure Chemicals, Tokyo, Japan). Motility was assessed by the hanging drop method [5]. Catalase and oxidase activities were determined using 3% hydrogen peroxide and cytochrome oxidase test strips (Nissui, Tokyo, Japan), respectively. For assessment of oxygen requirements, strain THUN1379ᵀ was cultured on 5% sheep blood agar (Becton Dickinson) and chocolate agar (Becton Dickinson) plates under aerobic and anaerobic conditions.

Colony morphology was examined following growth of the strain on R2A agar at 30 °C for 3 days. Cell morphology, size, and flagellar structure were further investigated by transmission electron microscopy. Cells grown on R2A agar were negatively stained with 2% (*w*/*v*) uranyl acetate and examined using a JEOL JEM-1400Flash transmission electron microscope operated at an accelerating voltage of 100 kV [6].

Biochemical characteristics were assessed using the API 20 EN system (bioMérieux Japan, Tokyo, Japan) and API ZYM system (bioMérieux), and the MicroScan WalkAway 40SI and Neg Combo EN 2J panels (Beckman Coulter, Tokyo, Japan) according to the manufacturers’ protocols. Growth under different temperatures (10–42 °C), pH values (pH 5.0–9.0), and NaCl concentrations (0–6%, *w*/*v*) was tested in tryptic soy broth. Colony morphology and growth were examined on TSA, R2A agar, blood agar, and MacConkey agar.

Cellular fatty acid profiling of THUN1379^T^ was performed using biomass harvested after cultivation on R2A agar at 30 °C for 72 h and analyzed with the Sherlock Microbial Identification System (MIDI Inc., Newark, DE, USA) according to the manufacturer’s instructions. Fatty acid peaks were identified using the TSBA6 library [7].

### 2.3. Whole-Genome Sequencing and Phylogenetic/Genomic Analysis

The genomic DNA of strain THUN1379^T^ was extracted from cultures grown on tryptic soy agar using the PowerSoil Pro Kit (Qiagen, Hilden, Germany), following the manufacturer’s instructions. Whole-genome sequencing was performed using a hybrid approach combining Oxford Nanopore long-read and Illumina short-read technologies [8,9]. For Nanopore sequencing, libraries were prepared using the Ligation Sequencing Kit SQK-LSK109 and Native Barcoding Expansion Kit EXP-NBD104, and sequencing was conducted on a MinION device with an R9.4.1 flow cell (Oxford Nanopore Technologies, Oxford, UK). Adapter trimming and quality filtering of Nanopore reads were performed using Porechop version 0.2.4 and Trim Galore version 0.6.7, respectively. For Illumina sequencing, libraries were prepared with the Nextera DNA Sample Preparation Kit and Nextera XT Index Kit v2, and sequencing was performed on a MiSeq platform (Illumina, San Diego, CA, USA) using the MiSeq Reagent Kit v3 (300 bp paired-end reads). Low-quality reads were trimmed using Trimmomatic version 0.38.1. Hybrid genome assembly was performed with Unicycler version 0.5.0 using both Nanopore and Illumina reads. Genome assembly statistics were obtained using QUAST version 5.3.1, and genome completeness and contamination were assessed using CheckM version 1.2.4 with lineage-specific marker gene sets. Default parameters were applied for all analyses.

From the assembled genome, the 16S rRNA gene sequence was extracted and used for phylogenetic analysis [10]. Multiple sequence alignment and phylogenetic tree construction were conducted using MEGA11 software version 11.0.13, and a neighbor-joining tree was generated with bootstrap values calculated from 1000 replicates [10]. For whole-genome-based phylogenetic analysis, the assembled genome of strain THUN1379^T^ and those of closely related taxa were analyzed using GTDB-Tk version 2.6.0 to infer phylogenomic placement based on concatenated marker genes. A maximum-likelihood phylogenetic tree was constructed using IQ-TREE, and branch support was assessed using bootstrap analysis. The resulting phylogenetic tree was visualized and annotated using the Interactive Tree Of Life (iTOL) web server [11,12].

Genomic relatedness was further assessed by calculating average nucleotide identity (ANI) values using the EZBioCloud ANI calculator and digital DNA–DNA hybridization (dDDH) values using the Type Strain Genome Server (TYGS) [13,14].

Putative virulence factor-related genes were screened using ABRicate version 1.0.1 with the Virulence Factor Database (VFDB) [15]. Screening was first performed using stringent thresholds (≥80% amino acid identity and ≥80% coverage), and no hits were detected. Additional screening was then performed using relaxed thresholds (≥70% identity and ≥60% coverage), and hits were evaluated based on identity and coverage.

### 2.4. MALDI-TOF MS Analysis

MALDI-TOF MS was performed using a Microflex LT system (Bruker Daltonics, Bremen, Germany). *P. flexuosum* sp. nov. THUN1379ᵀ and *P. purpuratum* KJ031ᵀ were cultured on R2A agar or tryptic soy agar at 30 °C for 24 h. Bacterial proteins were extracted from colonies using the ethanol-formic acid extraction protocol [16]. For each strain, spectra were obtained from three independent measurements. Calibration was carried out using the Bruker Bacterial Test Standard (Bruker Daltonics). Spectra were acquired and analyzed with the MALDI Biotyper 3.0 software (Bruker Daltonics). For detailed comparison of spectral profiles and peak analysis, FlexAnalysis version 3.4 and ClinProTools version 3.0 (Bruker Daltonics) were used [17,18,19]. Spectral comparison was conducted between *P. flexuosum* sp. nov. THUN1379ᵀ and *P. purpuratum* KJ031ᵀ, the phylogenetically closest related species.

## 3. Clinical Context

Strain THUN1379ᵀ was isolated from blood cultures obtained from a 78-year-old male patient who was diagnosed with pyogenic spondylitis at Takarazuka City Hospital, Japan. At admission, the patient presented with fever, and two sets of blood cultures were obtained. All four blood culture bottles subsequently became positive for the same Gram-negative curved rod-shaped bacterium. The anaerobic bottles became positive approximately 15 h after incubation, followed by the aerobic bottles at approximately 21 h.

Empirical antimicrobial therapy with intravenous ceftriaxone was initiated after blood culture collection and was subsequently changed to intravenous sulbactam/ampicillin based on Gram-stain findings and clinical suspicion. After the diagnosis of pyogenic spondylitis was established, antimicrobial therapy was switched to oral levofloxacin, which was continued during the outpatient period. The patient showed gradual clinical improvement with resolution of fever and symptoms.

Although bacterial culture from a lumbar spine biopsy specimen was negative, likely due to prior antimicrobial exposure, the presence of the organism in the lesion was later confirmed by 16S rRNA gene sequence analysis of formalin-fixed, paraffin-embedded tissue. A detailed description of the clinical course, antimicrobial treatment, and diagnostic workup has been reported previously in a peer-reviewed case report [20].

## 4. Results and Discussion

### 4.1. Phenotypic and Chemotaxonomic Characteristics

The distinguishing phenotypic characteristics of *P. flexuosum* sp. nov. THUN1379ᵀ in comparison with the type strains of other *Paludibacterium* species are summarized in Table 1.

**Figure 1:**
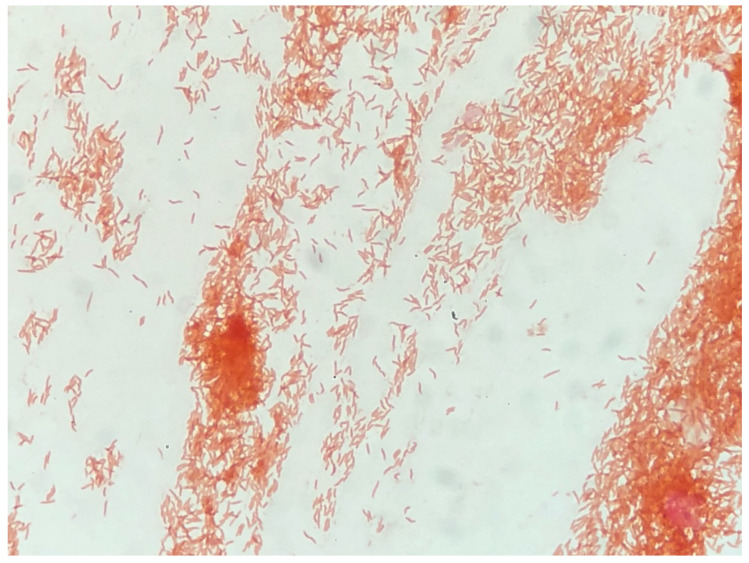
Gram-stained cells of *P. flexuosum* sp. nov. THUN1379^T^ showing Gram-negative, curved rod-shaped morphology.

*P. flexuosum* sp. nov. THUN1379ᵀ formed circular, white to translucent colonies with a diameter of approximately 1–1.5 mm after 24 h of incubation on tryptic soy agar, R2A agar, and 5% sheep blood agar at 35 °C under both aerobic and 5% CO_2_ conditions. Cells were Gram-negative, curved rod-shaped, motile, non-spore-forming, and lacked visible pigmentation (Figure 1).

**Figure 2:**
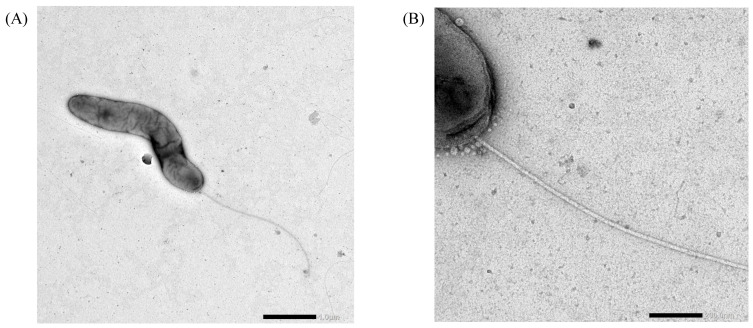
Transmission electron micrographs of *P. flexuosum* sp. nov. THUN1379^T^ grown for 72 h at 30 °C on R2A agar. (**A**) Whole-cell morphology showing a curved rod-shaped cell with a single polar flagellum. (**B**) Enlarged view of the polar region highlighting the single polar flagellum. Scale bars: 1.0 µm (**A**) and 200 nm (**B**).

Transmission electron microscopy further revealed curved rod-shaped cells equipped with a single polar flagellum, confirming a motile phenotype with a flagellar structure consistent with those previously reported for members of the genus *Paludibacterium* (Figure 2A,B). These morphological features are consistent with those reported for previously described members of the genus.

*P. flexuosum* sp. nov. THUN1379ᵀ was positive for oxidase activity, indole production, growth on 5% sheep blood agar, and L-ornithine decarboxylase activity, but negative for catalase activity. Notably, strain THUN1379ᵀ consistently showed negative catalase activity, despite catalase positivity being described as a typical characteristic of the genus *Paludibacterium*. To verify this result, catalase activity was re-evaluated using colonies grown on R2A agar for 48 h and tested with 3% hydrogen peroxide; all repeated tests yielded negative results. In addition, the type strain *P. purpuratum* KJ031ᵀ obtained from the Korean Collection for Type Cultures (KCTC) was examined under identical conditions, and catalase activity was also found to be negative, although the original species description reported catalase positivity. This discrepancy may reflect methodological differences, growth conditions, strain variability, or instability of catalase expression, which has been reported for several Gram-negative environmental bacteria. These findings suggest that catalase activity may not be a strictly conserved trait within the genus *Paludibacterium* and should be interpreted with caution in taxonomic characterization. This biochemical profile clearly differentiates *P. flexuosum* sp. nov. THUN1379ᵀ from its closest phylogenetic relative, *P. purpuratum* KJ031ᵀ, which exhibits distinct enzymatic reactions and carbohydrate utilization patterns (Table 1).

Cellular fatty acid analysis revealed that the major fatty acids of *P. flexuosum* sp. nov. THUN1379ᵀ were summed feature 3 (C16:1 ω7c and/or C16:1 ω6c), C16:0, and summed feature 8 (C18:1 ω7c and/or C18:1 ω6c), a profile consistent with other *Paludibacterium* species (Table 2). Notably, *P. flexuosum* sp. nov. THUN1379ᵀ exhibited slightly higher proportions of C12:0 and lower proportions of C16:0 compared with *P. purpuratum* KJ031ᵀ. Although these differences are subtle, they further support the chemotaxonomic distinction of *P. flexuosum* sp. nov. THUN1379ᵀ at the species level.

### 4.2. Phylogenetic and Genomic Analyses

**Figure 3:**
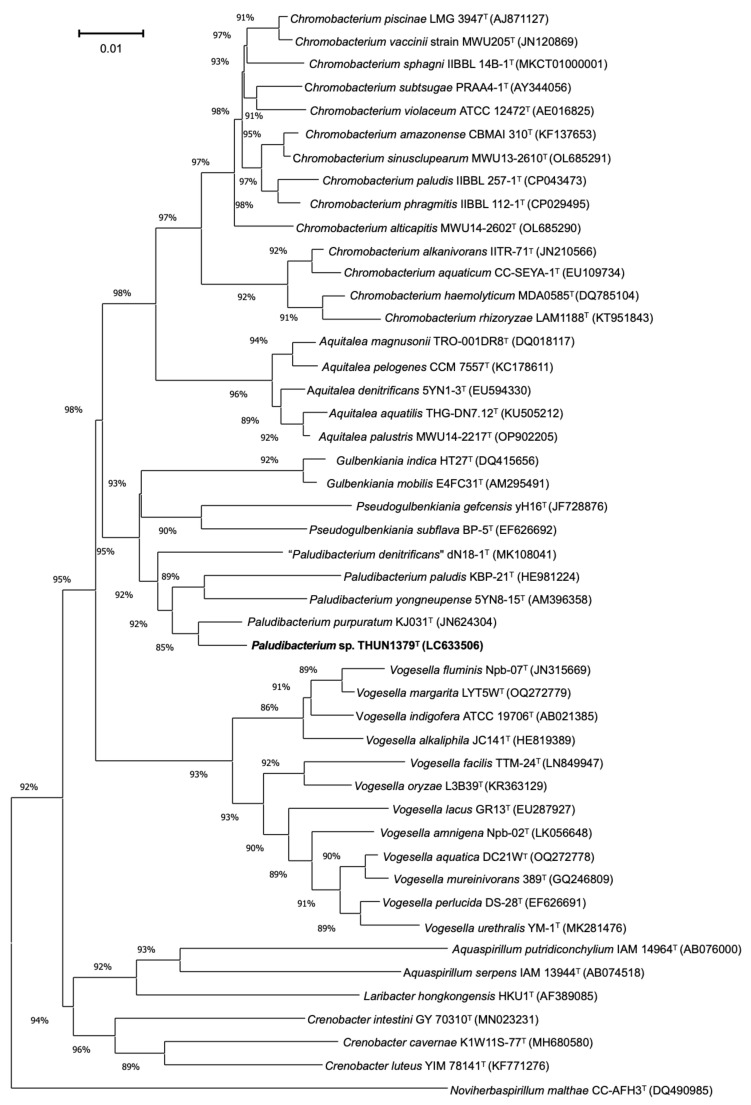
**Neighbour**-joining phylogenetic tree, based on 16S rRNA gene sequences, showing the phylogenetic positions of *P. flexuosum* sp. nov. THUN1379^T^ and other closely related members of the family *Chromobacteriaceae*. Numbers at nodes are bootstrap values (% of 1000 replicates). *Noviherbaspirillum malthae* CC-AFH3^T^ was used as an outgroup. The scale bar represents 0.01 substitutions per site.

The nearly complete 16S rRNA gene sequence of *P. flexuosum* sp. nov. THUN1379ᵀ showed highest sequence similarity to *P. purpuratum* KJ031ᵀ (98.6%), followed by *P. paludis* KBP-21ᵀ (97.4%) and *P. yongneupense* 5YN8-15ᵀ (97.2%). In the neighbour-joining phylogenetic tree based on 16S rRNA gene sequences, *P. flexuosum* sp. nov. THUN1379ᵀ clustered within the genus *Paludibacterium* but formed a distinct and independent lineage separated from all currently recognized species (Figure 3).

**Figure 4:**
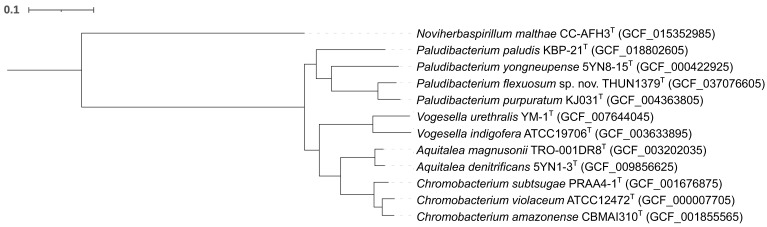
Maximum-likelihood phylogenetic tree, based on whole-genome sequences, showing the phylogenetic positions of *P. flexuosum* sp. nov. THUN1379^T^ and other closely related members. Numbers at nodes are bootstrap values (% of 1000 replicates). *Noviherbaspirillum malthae* CC-AFH3^T^ was used as an outgroup. The scale bar represents 0.1 substitutions per site.

To further resolve its taxonomic position, whole-genome-based phylogenetic analysis was performed. The hybrid assembly of *P. flexuosum* sp. nov. THUN1379ᵀ resulted in a high-quality genome with a total size of 3,613,774 bp. Genome completeness and contamination were estimated to be 99.57% and 0.45%, respectively, indicating suitability for phylogenomic analysis. In the maximum-likelihood phylogenomic tree inferred from concatenated conserved marker genes using GTDB-Tk and IQ-TREE, *P. flexuosum* sp. nov. THUN1379ᵀ formed a clearly distinct branch within the *Paludibacterium* clade, separate from *P. purpuratum*, *P. paludis*, and *P. yongneupense* (Figure 4). This topology was well supported by bootstrap values and was congruent with the 16S rRNA gene-based phylogeny.

Genomic relatedness analyses further supported species-level differentiation. The average nucleotide identity (ANI) value between *P. flexuosum* sp. nov. THUN1379ᵀ and *P. purpuratum* KJ031ᵀ was 79.7%, and the digital DNA-DNA hybridization (dDDH) value was 23.1%, both far below the accepted thresholds for species delineation (≥95–96% for ANI and ≥70% for dDDH). Comparable low ANI and dDDH values were observed between *P. flexuosum* sp. nov. THUN1379ᵀ and other *Paludibacterium* species (Table 3). Collectively, these genomic metrics unambiguously indicate that *P. flexuosum* sp. nov. THUN1379ᵀ represents a novel species within the genus *Paludibacterium*.

Notably, the isolate described in this study was previously reported as *P. purpuratum* based solely on 16S rRNA gene sequence analysis in a clinical case report [20]. However, subsequent whole-genome-based analyses clearly demonstrated that strain THUN1379ᵀ represents a distinct genomic lineage, warranting its classification as a novel species within the genus *Paludibacterium*. To date, reports of human infections caused by members of the genus *Paludibacterium* are extremely limited. In addition to the previously reported Japanese case of bacteremia associated with pyogenic spondylitis [20], only one further clinical case has been described in the literature. In that report, *P. purpuratum* was identified as the causative agent of severe pneumonia complicated by septicemia in an elderly patient in Taiwan [21]. Both clinical reports indicate that *Paludibacterium* species, although originally considered environmental bacteria, can cause invasive infections, including bloodstream infections, in susceptible hosts. Together with the present genomic re-evaluation, these findings suggest that clinically isolated strains previously identified as *P. purpuratum* based solely on 16S rRNA gene sequence analysis may represent distinct lineages within the genus.

### 4.3. Virulence Factor-Related Gene Analysis

Genome-based screening of *P. flexuosum* sp. nov. THUN1379ᵀ was performed using ABRicate with the Virulence Factor Database (VFDB) to explore the presence of putative virulence factor-related genes. When stringent default criteria commonly applied in VFDB-based analyses (≥80% amino acid identity and ≥80% coverage) were used, no virulence factor-related genes were detected in *P. flexuosum* sp. nov. THUN1379ᵀ. However, when less stringent thresholds (≥70% identity and ≥60% coverage) were applied, several genes annotated as putative virulence factor-related genes were identified (Table 4 and Supplementary Table S1). The relaxed thresholds were applied to explore distant homologs that may be associated with general cellular processes rather than to identify confirmed virulence determinants. Accordingly, hits detected under these conditions should be interpreted as putative homologs and not as evidence of preserved virulence-related functions. This approach was adopted to provide a broader overview of potential functional relationships while avoiding overinterpretation of virulence potential. Because sequence identity levels of approximately 70% do not necessarily indicate functional conservation, these results were not regarded as direct evidence of pathogenicity.

Under these relaxed criteria, two genes, *bplF* and *xcpR*, were detected exclusively in *P. flexuosum* sp. nov. THUN1379ᵀ among the analyzed *Paludibacterium* species (Table 4). The *bplF* gene encodes a putative lipopolysaccharide biosynthesis-related protein, which is annotated in VFDB based on *Bordetella pertussis*. Lipopolysaccharide-associated genes are widely distributed among Gram-negative bacteria and are primarily involved in cell envelope biogenesis and structural integrity rather than direct virulence [22,23]. The moderate sequence identity (70.5%) and coverage (60.8%) observed for *bplF* in *P. flexuosum* sp. nov. THUN1379ᵀ suggest distant homology rather than functional equivalence to the *Bordetella* virulence factor, and its presence is more likely related to general lipopolysaccharide biosynthesis.

The *xcpR* gene, annotated as a general secretion pathway protein of the type II secretion system (T2SS), showed 72.3% identity and 63.2% coverage in *P. flexuosum* sp. nov. THUN1379ᵀ and was not detected in other *Paludibacterium* type strains under the same criteria. The T2SS is known to mediate the secretion of extracellular enzymes and toxins in various Gram-negative pathogens; however, it is also commonly present in environmental bacteria, where it contributes to nutrient acquisition and ecological adaptation. In our VFDB-based screening, no additional core components indicative of a complete and functional T2SS were identified, suggesting that *xcpR* alone is unlikely to confer classical virulence but may instead represent a secretion-related element with limited or ancestral functionality [24,25].

### 4.4. MALDI-TOF MS Results

MALDI-TOF MS analysis was performed to evaluate proteomic relatedness between *P. flexuosum* sp. nov. THUN1379ᵀ and closely related taxa. For each strain, spectra were obtained from three independent measurements. *P. flexuosum* sp. nov. THUN1379ᵀ could not be reliably identified using the MALDI Biotyper reference database, which currently lacks spectra for members of the genus *Paludibacterium*. The highest identification score obtained was 1.214 for *Comamonas kerstersii*, indicating the absence of a close match in the database.

**Figure 5:**
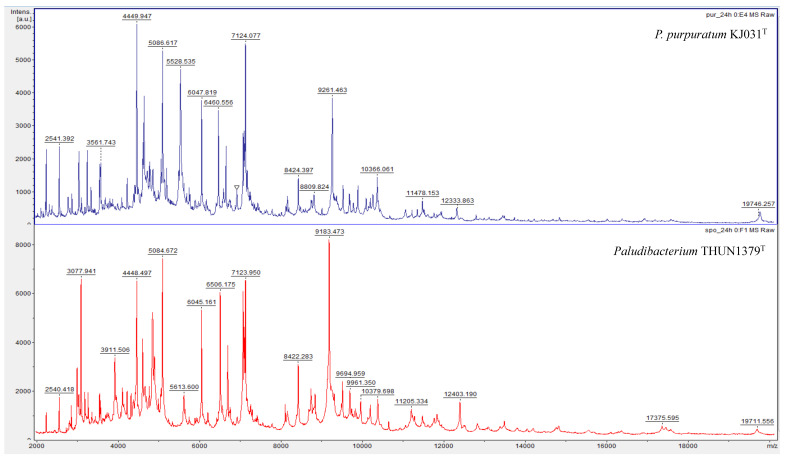
MALDI-TOF MS spectra overall patterns of *P. purpuratum* KJ031^T^ and *P. flexuosum* sp. nov. THUN1379^T^ with the highest similarity in whole genome sequence. *P. purpuratum* KJ031^T^ at the **top** and *P. flexuosum* sp. nov. THUN1379^T^ at the **bottom**.

**Figure 6:**
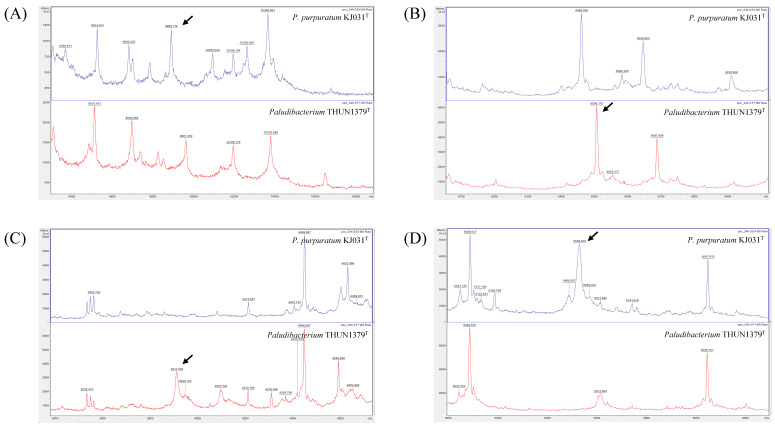
Spectral peaks to differentiate *P. flexuosum* sp. nov. THUN1379^T^ and *P. purpuratum* KJ031^T^ by MALDI-TOF MS. *P. purpuratum* KJ031^T^ at the top and *P. flexuosum* sp. nov. THUN1379^T^ at the bottom; (**A**) around 9893 *m*/*z*, (**B**) around 6508 *m*/*z*, (**C**) around 3913 *m*/*z*, and (**D**) around 5531 *m*/*z*.

Direct comparison of MALDI-TOF MS spectra between *P. flexuosum* sp. nov. THUN1379ᵀ and *P. purpuratum* KJ031ᵀ revealed clear differences in overall spectral patterns (Figure 5). Several strain-specific peaks were observed at approximately *m*/*z* 9893, 6508, 3913, and 5531, which were consistently present in *P. flexuosum* sp. nov. THUN1379ᵀ but absent or shifted in *P. purpuratum* KJ031ᵀ across replicate analyses. These discriminatory peaks were identified by spectral peak analysis using ClinProTools software, and the corresponding results are summarized in Table 5 (Figure 6). These reproducible proteomic differences further support the differentiation of *P. flexuosum* sp. nov. THUN1379ᵀ from its closest phylogenetic relative.

### 4.5. Description of Paludibacterium flexuosum sp. nov.

*Paludibacterium flexuosum* (fle.xu.o’sum. L. neut. adj. flexuosum, sinuous or flexuous, referring to the curved and slightly flexuous morphology of the cells).

Cells are Gram-negative, curved rod-shaped, motile, and non-spore-forming. Motility is conferred by a single polar flagellum, as observed by transmission electron microscopy. Cells are facultatively anaerobic. Colonies formed on tryptic soy agar, R2A agar, and 5% sheep blood agar after 24 h of incubation at 35 °C under aerobic or 5% CO_2_ conditions are circular, smooth, white to translucent, and approximately 1.0–1.5 mm in diameter. Growth occurs at temperatures ranging from 25 to 42 °C, at pH 5.0–8.0, and in the presence of up to 1.5% (*w*/*v*) NaCl.

The strain is positive for oxidase activity, indole production, growth on 5% sheep blood agar, and L-ornithine decarboxylase activity, but negative for catalase activity. Catalase negativity was consistently observed in repeated assays under standardized growth conditions. Biochemical characteristics further differentiate the species from other members of the genus *Paludibacterium*. The major cellular fatty acids are summed feature 3 (C16:1 ω7c and/or C16:1 ω6c), C16:0, and summed feature 8 (C18:1 ω7c and/or C18:1 ω6c).

The type strain, THUN1379ᵀ, was isolated from blood cultures obtained from a patient with pyogenic spondylitis at Takarazuka City Hospital, Japan. The presence of the organism in a lumbar spine biopsy specimen from the same patient was subsequently confirmed by 16S rRNA gene sequence analysis of formalin-fixed, paraffin-embedded tissue. The genome of strain THUN1379ᵀ comprises approximately 3.61 Mb, with a DNA G+C content of 61.3 mol%. The complete genome sequence of the type strain THUN1379ᵀ has been deposited in the public databases under the accession number AP029060. The type strain is deposited in the Japan Collection of Microorganisms (JCM 36560ᵀ) and the Korean Collection for Type Cultures (KCTC 8465ᵀ).

## Figures and Tables

**Table 1:** Differential characteristics of *P. flexuosum* sp. nov. THUN1379^T^ and the type strains of other species of the genus *Paludibacterium*. Data compiled from reference number 3 and the current study.

Characteristic	*P. flexuosum* sp. nov. THUN1379^T^	*P. purpuratum * KJ031^T^	*P. paludism * KBP-21^T^	*P. yongneupense * 5YN8-15^T^
Colony color (R2A medium)	White or translucent	Purple	Yellow	Cream
Oxidase	+	+	+	+
Catalase	− *^a^*	+ or − *^a^*	+	+
Temperature range for growth (°C)	25–42	20–37	15–42	15–37
NaCl concn. Range for growth (%)	0–1.5	0–1.5	0–2	0–0.5
pH range for growth	5–8	6–8	6–8	7–8
Growth on MacConkey agar	−	−	+	+
Growth on Blood agar	+	−	ND	ND
API 20NE results				
Gelatin liquefaction	−	+	+	−
Nitrate reduction	+	−	−	+
L-tryptophan (indole)	+	−	−	−
Enzyme activities (API ZYM)				
Esterase lipase (C8)	+	+	+	−
Naphthol-AS-BI-phosphohydrolase	+	+	−	+
α-glucosidase, β-glucosidase	−	−	−	+
*N*-Acetyl-β-glucosaminidase	−	−	+	+
Microscan WalkAway results				
Indole	+	−	−	−
Ornithine	+	−	−	−
Antibiotics				
Ampicillin	R	R	R	S
Kanamycin	S	S	R	R

*^a^* Catalase activity was assessed using colonies grown on R2A agar for 48 h and tested by the bubble formation method with 3% (*v*/*v*) hydrogen peroxide. Under these conditions, both *P. flexuosum* THUN1379ᵀ and *P. purpuratum* KJ031ᵀ showed negative catalase activity, although catalase positivity has been reported for *P. purpuratum* in the original species description. This discrepancy may reflect differences in growth conditions or physiological states.

**Table 2:** Fatty acid compositions (as percentages of the total fatty acids present) of strain THUN1379^T^ and the type strains of the three species of the genus *Paludibacterium*.

Fatty Acid	1	2	3	4
C_10:0_ 3-OH	2.8	3.5	3.6	2.5
C_12:0_	10.19	7.3	6.8	9.5
C_12:0_ 2-OH	ND	1	ND	ND
C_12:0_ 3-OH	3.36	3.4	2.4	4.2
C_14:1_ *ω*5c	TR	TR	TR	1.1
C_14:0_	5.78	4.8	4.5	5.5
C_16:0_	21.77	27.8	23.9	35.9
C_17:1_ *ω*6c	2.4	1.5	1.4	ND
C_17:0_	TR	1.3	1.5	ND
C_17:0_ cyclo	ND	ND	19.8	ND
C_19:0_ *ω*8c cyclo	ND	ND	ND	1.8
Summed feature 3 *^a^*	30.59	28	29.9	10.7
Summed feature 8 *^a^*	18.66	18.4	21.7	6.2

Strains: 1, *Paludibacterium flexuosum* sp. nov. THUN1379^T^; 2, *P. purpuratum* KJ031^T^; 3, *P. paludis* KBP-21^T^; 4, *P. yongneupense* 5YN8-15^T^. The data of THUN1379^T^ are from the present study, and other data are from [3]. Only fatty acids amounting to at least 1.0% of the total fatty acids of at least one of the strains are shown. TR, trace (<1.0%); ND, not detected. *^a^* Summed Feature is the sum of all fatty acids for which the fatty acid species cannot be identified because the Retention Time and ECL values are almost the same in the MIDI method, including peaks such as their degradation products. Summed feature 3 was listed as C_16:1_ *ω*7c and/or C_16:1_ *ω*6c. Summed feature 8 was listed as C_18:1_ *ω*7c and/or C_18:1_ *ω*6c.

**Table 3:** Comparison of *P. flexuosum* sp. nov. THUN1379^T^ 16S rRNA gene sequence and whole-genome sequence with type strains of *P. purpuratum* KJ031^T^, *P. paludis* KBP-21^T^, *P. yongneupense* 5YN8-15^T^, and “*P. denitrificans*” dN18-1^T^.

Genus *Paludibacterium*	*P. flexuosum* sp. nov. THUN1379^T^		
	16S rRNA Gene Sequence Similarity (%)	ANI (%)	dDDH (%)
*P. purpuratum* KJ031^T^	98.6	79.7	23.1
*P. paludis* KBP-21^T^	97.4	74.3	20.6
*P. yongneupense* 5YN8-15^T^	97.2	73.9	19.9
“*P. denitrificans*” dN18-1^T^	97	73.8	20.5

Species delineation thresholds followed established criteria (<98.7% 16S rRNA similarity, <95–96% ANI, and <70% dDDH) [13]. ANI, average nucleotide identity; dDDH, digital DNA–DNA hybridization.

**Table 4:** Virulence factor-related genes detected in *P. flexuosum* sp. nov. THUN1379^T^ and genus *Paludibacterium* by VFDB screening.

Gene (VFDB Annotation)	Putative Function	% Identity	% Coverage	*P. flexuosum* sp. nov. THUN1379^T^	*P. purpuratum* KJ031^T^	*P. paludis* KBP-21^T^	*P. yongneupense* 5YN8-15^T^
*bplF*	LPS biosynthesis-related protein	70.5	60.8	+	–	–	–
*xcpR*	Type II secretion system protein	72.3	63.2	+	–	–	–

**Table 5:** Comparison of MALDI-TOF MS mass spectra of *P. flexuosum* sp. nov. THUN1379^T^ and *P. purpuratum* KJ031^T^, the most similar whole genome sequence.

Mass (*m*/*z*)	*p* Value	Peak Area/Intensity Average *^a^* of *P. flexuosum* sp. nov. THUN1379^T^	Peak Area/Intensity Average *^a^* of *P. purpuratum* KJ031^T^
9893	<0.001	0.48	5.71
6508	0.044	22.89	1.41
3913	0.044	11.87	0.09
5531	0.047	0.97	22.13

*^a^* Each strain was analyzed in triplicate (three independent MALDI-TOF MS measurements per strain). Peak area/intensity values represent the average of three measurements.

## Data Availability

The complete genome sequence of *Paludibacterium flexuosum* sp. nov. THUN1379ᵀ has been deposited in GenBank under accession number AP029060. The 16S rRNA gene sequence is available under accession number LC633506. Raw sequencing data generated by Illumina and Oxford Nanopore sequencing have been deposited in DDBJ Sequence Read Archive (DRA) under accession numbers DRR900256 (Illumina) and DRR 900257 (Oxford Nanopore).

## Supplementary Materials

The following supporting information can be downloaded at: https://www.mdpi.com/article/10.3390/microorganisms14020280/s1, Table S1: Virulence factor–related genes identified by VFDB screening in *P. flexuosum* sp. nov. THUN1379T and genus *Paludibacterium* using ABRicate.

## References

[1] Kwon S.W., Kim B.Y., Weon H.Y., Baek Y.K., Yoo S.H., Koo B.S., Go S.J. (2008). *Paludibacterium yongneupense* gen. nov., sp. nov., isolated from a wetland, Yongneup, in Korea. Int. J. Syst. Evol. Microbiol..

[2] Sheu S.Y., Chen Z.H., Young C.C., Chen W.M. (2014). *Paludibacterium paludis* sp. nov., isolated from a marsh. Int. J. Syst. Evol. Microbiol..

[3] Kang H., Kim H., Joung Y., Kim K.J., Joh K. (2016). *Paludibacterium purpuratum* sp. nov., isolated from wetland soil. Int. J. Syst. Evol. Microbiol..

[4] Lee J.E., Park S., Kang H., Kim H., Joh K. (2022). *Paludibacterium denitrificans* sp. nov., a novel denitrifying bacterium isolated from activated sludge. Curr. Microbiol..

[5] Brown A.E. (2007). Benson’s Microbiological Applications: Laboratory Manual in General Microbiology.

[6] Brenner S., Horne R.W. (1959). A negative staining method for high resolution electron microscopy of viruses. Biochim. Biophys. Acta.

[7] Sasser M. (1990). Identification of Bacteria by Gas Chromatography of Cellular Fatty Acids.

[8] Nakahara T., Nakamura A., Tamai K., Komatsu M., Ohkusu K. (2025). *Rodentibacter abscessus* sp. nov. isolated from pet hamster subcutaneous abscess. Int. J. Syst. Evol. Microbiol..

[9] Nakamura A., Nakamura T., Niki M., Kuchibiro T., Nishi I., Komatsu M. (2021). Genomic characterization of ESBL- and carbapenemase-positive Enterobacteriaceae co-harboring mcr-9 in Japan. Front. Microbiol..

[10] Saito H., Iwamoto T., Ohkusu K., Otsuka Y., Akiyama Y., Sato S., Taguchi O., Sueyasu Y., Kawabe Y., Fujimoto H. (2011). *Mycobacterium shinjukuense* sp. nov., a slowly growing, non-chromogenic species isolated from human clinical specimens. Int. J. Syst. Evol. Microbiol..

[11] Nguyen L.-T., Schmidt H.A., von Haeseler A., Minh B.Q. (2015). IQ-TREE: A fast and effective stochastic algorithm for estimating maximum-likelihood phylogenies. Mol. Biol. Evol..

[12] Letunic I., Bork P. (2019). Interactive Tree Of Life (iTOL) v4: Recent updates and new developments. Nucleic Acids Res..

[13] Goris J., Konstantinidis K.T., Klappenbach J.A., Coenye T., Vandamme P., Tiedje J.M. (2007). DNA–DNA hybridization values and their relationship to whole-genome sequence similarities. Int. J. Syst. Evol. Microbiol..

[14] Auch A.F., von Jan M., Klenk H.P., Göker M. (2010). Digital DNA–DNA hybridization for microbial species delineation by means of genome-to-genome sequence comparison. Stand. Genomic Sci..

[15] Chen L., Yang J., Yu J., Yao Z., Sun L., Shen Y., Jin Q. (2005). VFDB: A reference database for bacterial virulence factors. Nucleic Acids Res..

[16] Seng P., Drancourt M., Gouriet F., La Scola B., Fournier P.E., Rolain J.M., Raoult D. (2009). Ongoing revolution in bacteriology: Routine identification of bacteria by matrix-assisted laser desorption ionization time-of-flight mass spectrometry. Clin. Infect. Dis..

[17] Kubo Y., Ueda O., Nagamitsu S., Yamanishi H., Nakamura A., Komatsu M. (2021). Novel strategy of rapid typing of Shiga toxin-producing *Escherichia coli* using MALDI Biotyper and ClinProTools analysis. J. Infect. Chemother..

[18] Nakamura A., Komatsu M., Kondo A., Ohno Y., Kohno H., Nakamura F., Matsuo S., Ohnuma K., Hatano N., Kawano S. (2015). Rapid detection of B2-ST131 clonal group of extended-spectrum β-lactamase-producing *Escherichia coli* by matrix-assisted laser desorption ionization-time-of-flight mass spectrometry: Discovery of a peculiar amino acid substitution in B2-ST131 clonal group. Diagn. Microbiol. Infect. Dis..

[19] Nakamura A., Komatsu M., Ohno Y., Noguchi N., Kondo A., Hatano N. (2019). Identification of specific protein amino acid substitutions of extended-spectrum β-lactamase (ESBL)-producing *Escherichia coli* ST131: A proteomics approach using mass spectrometry. Sci. Rep..

[20] Murakami J., Murakami M., Nakamura A., Yamada K., Oka K., Yamagishi Y., Mikamo H. (2022). A case of pyogenic spondylitis caused by *Paludibacterium purpuratum*. J. Infect. Chemother..

[21] Cia C.T., Chen J.W., Su S.L., Tsai P.F., Shu C.Y., Ko W.C., Chen P.L. (2021). Severe Lung Infection and Septicemia Caused by *Paludibacterium purpuratum*—A Case Report and Evaluation of Bacterial Traits. Open Forum Infect. Dis..

[22] Raetz C.R.H., Whitfield C. (2002). Lipopolysaccharide endotoxins. Annu. Rev. Biochem..

[23] Allen A., Maskell D.J. (1996). Identification, cloning and mutagenesis of a genetic locus required for lipopolysaccharide biosynthesis in Bordetella pertussis. Mol. Microbiol..

[24] Sandkvist M. (2001). Biology of type II secretion. Mol. Microbiol..

[25] Cianciotto N.P. (2005). Type II secretion: A protein secretion system for all seasons. Trends Microbiol..

